# Leptolysin, a *Leptospira* secreted metalloprotease of the pappalysin family with broad-spectrum activity

**DOI:** 10.3389/fcimb.2022.966370

**Published:** 2022-08-23

**Authors:** Daniella dos Santos Courrol, Cristiane Castilho Fernandes da Silva, Luan Gavião Prado, Rosa Maria Chura-Chambi, Ligia Morganti, Gisele Oliveira de Souza, Marcos Bryan Heinemann, Lourdes Isaac, Fernando Paiva Conte, Fernanda Calheta Vieira Portaro, Rodrigo Nunes Rodrigues-da-Silva, Angela Silva Barbosa

**Affiliations:** ^1^ Laboratory of Bacteriology, Butantan Institute, São Paulo, Brazil; ^2^ Laboratory of Structure and Function of Biomolecules, Butantan Institute, São Paulo, Brazil; ^3^ Department of Microbiology, Institute of Biomedical Sciences, University of São Paulo, São Paulo, Brazil; ^4^ Center of Biotechnology, Energy and Nuclear Research Institute (IPEN)-CNEN/SP), São Paulo, Brazil; ^5^ Department of Preventive Veterinary Medicine and Animal Health, School of Veterinary Medicine and Animal Science, University of São Paulo, São Paulo, Brazil; ^6^ Department of Immunology, Institute of Biomedical Sciences, University of São Paulo, São Paulo, Brazil; ^7^ Pilot Plant Implementation Project, Immunobiological Technology Institute, Oswaldo Cruz Foundation, Rio de Janeiro, Brazil; ^8^ Laboratory of Immunological Technology, Immunobiological Technology Institute, Oswaldo Cruz Foundation, Rio de Janeiro, Brazil

**Keywords:** leptolysin, pappalysin-1 domain protein, *Leptospira*, proteolytic activity, extracellular matrix degradation

## Abstract

Extracellular proteolytic enzymes are produced by a variety of pathogenic microorganisms, and contribute to host colonization by modulating virulence. Here, we present a first characterization of leptolysin, a *Leptospira* metalloprotease of the pappalysin family identified in a previous exoproteomic study. Comparative molecular analysis of leptolysin with two other pappalysins from prokaryotes, ulilysin and mirolysin, reveals similarities regarding calcium, zinc, and arginine -binding sites conservation within the catalytic domain, but also discloses peculiarities. Variations observed in the primary and tertiary structures may reflect differences in primary specificities. Purified recombinant leptolysin of *L. interrogans* was obtained as a ~50 kDa protein. The protease exhibited maximal activity at pH 8.0 and 37°C, and hydrolytic activity was observed in the presence of different salts with maximum efficiency in NaCl. Substrate specificity was assessed using a small number of FRET peptides, and showed a marked preference for arginine residues at the P1 position. *L. interrogans* leptolysin proteolytic activity on proteinaceous substrates such as proteoglycans and plasma fibronectin was also evaluated. All proteins tested were efficiently degraded over time, confirming the protease´s broad-spectrum activity *in vitro*. In addition, leptolysin induced morphological alterations on HK-2 cells, which may be partially attributed to extracellular matrix (ECM) degradation. Hemorrhagic foci were observed in the dorsal skin of mice intradermally injected with leptolysin, as a plausible consequence of ECM disarray and vascular endothelium glycocalyx damage. Assuming that leptospiral proteases play an important role in all stages of the infectious process, characterizing their functional properties, substrates and mechanisms of action is of great importance for therapeutic purposes.

## 1 Introduction

Spirochetes of the genus *Leptospira* are widespread microorganisms which can be found in the environment as free-living bacteria or infect susceptible hosts causing disease. To date 66 *Leptospira* species have been described and classified into 4 subclades. The P1 and P2 subclades comprise the pathogenic species. While species within the P1 subclade are more frequently associated to severe human disease, those within the P2 subclade have a lower level of pathogenicity, and generally cause mild or even asymptomatic illness. The S1 and S2 subclades encompass saprophytic species (Vincent et al., 2019; Caimi and Ruybal, 2020; Casanovas-Massana et al., 2021).

Pathogenic *Leptospira* are extremely efficient in colonizing human and animal hosts. Their high motility capacity associated to the ability to escape hosts´ innate immune responses ensures rapid dissemination and colonization of target organs (Wunder et al., 2016; Barbosa and Isaac, 2020). Additionally, leptospires are capable of degrading components of the ECM and plasma, and those mechanisms may contribute to bacterial invasion and immune evasion within the host (Fraga et al., 2014; da Silva et al., 2018).

The mechanisms underlying tissue damage caused by pathogenic leptospires are still poorly understood, but extracellular *Leptospira* proteases seem to play a role in helping these spirochetes to cross the body’s physical barriers. Collagen-degrading enzymes such as ColA are produced by pathogenic leptospires, and were shown to facilitate transcytosis through endothelial and renal cell monolayers (Kassegne et al., 2014). Leptallo I, another leptospiral metalloprotease of the M23 family, presents elastinolytic activity which may affect lung and endothelial function (Hashimoto et al., 2013). Extracellular proteases may also contribute to *Leptospira* survival in the bloodstream by inactivating effector proteins of the complement system as well as the antimicrobial peptide cathelicidin (Fraga et al., 2014; Oliveira et al., 2021). An example is the thermolysin encoded by *LIC13322*, which presents proteolytic activity against complement components C3 and C6 (Amamura et al., 2017; Chura-Chambi et al., 2018). In addition, M16-type metallopeptidases are involved in *Leptospira* invasiveness and transmission (Ge et al., 2020).

During infection, leptospires cross epithelial and endothelial barriers to reach target organs. Human endothelial cells infected with *L. interrogans* display marked morphological alterations that can be mostly attributed to intercellular junction disruption (Sato and Coburn, 2017). In *Leptospira*-infected renal epithelial cells, disassembly of the apical junction complex in epithelial cells allows transmigration of bacteria through the paracellular route (Sebastián et al., 2021). It is plausible to assume that bacterial protease-mediated cleavage of cell-cell junctions may be one of the factors that compromise tissue integrity.

We have previously shown that *L. interrogans* secretes proteases that degrade ECM and plasma proteins from the host (da Silva et al., 2018). Exoproteome analysis allowed identification of a 50 kDa metalloprotease that harbors a pappalysin-1 domain. It belongs to the M43 family and contains a HEXXHXXGXXH zinc-dependent active site. The human orthologue (“pregnancy-associated plasma protein-A” or PAPP-A) was nominated the first member of the pappalysin family of metallopeptidases (Boldt et al., 2001). Pappalysins have a crucial role in growth and development by mediating hydrolysis of insulin-like growth factor binding proteins (Oxvig, 2015). Putative PAPP-A homologues are categorized in two main groups: short pappalysins from bacteria, archaea and fungi consisting of a catalytic domain and a pro-domain, and large pappalysins which are multi-domain proteins found in vertebrates (Tallant et al., 2006; García-Castellanos et al., 2007). To our knowledge, only two short pappalysins have been characterized in detail to date: the protease ulilysin from the bacterium *Methanosarcina acetivorans*, and mirolysin, another LysargiNase from *Tannerella forsythia* (Tallant et al., 2006; García-Castellanos et al., 2007; Tallant et al., 2007; Huesgen et al., 2014; Jusko et al., 2015; Koneru et al., 2017; Guevara et al., 2020; Zak et al., 2021). Ulilysin is active against several substrates including casein, azoalbumin and ECM components. Furthermore, it has gelatinolytic activity and degrades elastin, actin and fibrinogen (Tallant et al., 2007). Mirolysin targets host proteins such as fibronectin, fibrinogen, complement proteins and the antimicrobial peptide LL-37 (Koneru et al., 2017). In light of the above considerations, and given that microbial proteases can potentially dismantle host structural and functional molecules to facilitate invasion and weaken the immune response, this work aimed to provide a first characterization of pappalysin-1 domain protein from *Leptospira* (henceforth called leptolysin) identified in our previous exoproteomic study (da Silva et al., 2018).

## 2 Materials and methods

### 2.1 Leptolysin three-dimensional (3D) structure modeling

The 3D structure of leptolysin was modeled using the Robetta server (new.robetta.org; accessed on 10 October 2021) (Kim et al., 2004; Baek et al., 2021), based on the full-length amino acid sequence of the protein (Uniprot ID Q72LV9) excluding its signal peptide (1-22). This server is continually evaluated through CAMEO (Continuous Automated Model Evaluation) and generates five models, that were analyzed by MolProbity (molprobity.biochem.duke.edu; accessed on 16 October 2021), a widely used system of model validation for protein structures (Williams et al., 2018). The best predictive model was selected and used in further analyzes. The protein ulilysin from *Methanosarcina acetivorans* (PDB: 3LUM) harbors a pappalysin-1 domain and was used for comparative modeling.

### 2.2 Prediction of leptolysin binding sites

Prediction of leptolysin calcium, zinc, and substrate binding sites was assessed with COACH (https://zhanggroup.org/COACH/), a meta-server approach that uses the combination of five methods (TM-SITE, S-SITE, including COFACTOR, FINDSITE, and ConCavity) to generate final ligand binding site predictions (Yang et al., 2013). Consensus binding residues predicted by COACH were compared with other M43 peptidases to evaluate their conservation among species.

### 2.3 Comparison of leptolysin with other members of the pappalysin family (M43B) of metalloproteases

Meg Align 4.0 was used to assess conservation of leptolysin residues within the predicted binding sites with those present in other metalloproteases. Leptolysin sequence (Uniprot accession number (UP): Q72LV9) was aligned by Muscle to sequences of: *Methanosarcina acetivorans* ulilysin (UP: Q8TL28), *Tannerella forsythia* mirolysin (UP: A0A0F7IPS1), *Arthroderma gypseum* extracellular metalloprotease MGYG_00389 (UP: E5QZI4), *Rhodococcus opacus* ulilysin (UP: A0A076EJ19), *Homo sapiens* Pappalysin-1 (UP: Q13219) and Pappalysin-2 (UP: Q9BXP8). *PyMol* was used to structurally align leptolysin, *M. acetivorans* ulilysin (Pdb: 3LUM and 2CKI) and *Tannerella forsythia* mature mirolysin (6R7W).

### 2.4 Evaluation of leptolysin conservation in *Leptospira* spp.

Leptolysin catalytic domain conservation among *Leptospira* species was assessed using Muscle (Edgar, 2004). *L. interrogans* serovar Copenhageni strain Fiocruz L1-130 leptolysin (a.a. 386 – 480 encompassing the zinc-dependent active site motif HEXXHXXGXXH) (UP: Q72LV9) was aligned and compared to leptolysin from sixteen pathogenic *Leptospira* species of the subclade P1: *L. kirschneri* (NCBI Reference Sequence (NCBI): WP_016753460.1), *L. noguchii* (NCBI: WP_020980541.1), *L. santarosai* (NCBI: WP_075979720.1), *L. mayottensis* (NCBI: WP_117339425.1), *L. borgpetersenii* (NCBI: WP_061220861.1), *L. alexanderi* (NCBI: WP_078128193.1), *L. weilii* (NCBI: WP_004508724.1), *L. alstonii* (NCBI: WP_020771835.1), *L. yasudae* (NCBI: WP_118954567.1), *L. barantonii* (WP_135669758.1), *L. kmetyi* (NCBI: WP_100737846.1), *L. tipperaryensis* (NCBI: WP_069608932.1), *L. stimsonii* (NCBI: WP_118980449.1), *L. adleri* (NCBI: WP_100784592.1), *L. ellisii* (NCBI: WP_100745687.1), *L. gomenensis* (NCBI: WP_135590981.1), six intermediate species of the subclade P2: *L. fainei* (NCBI: WP_016549876.1), *L. fluminis* (NCBI: WP_135812479.1), *L. fletcheri* (NCBI: WP_135767071.1), *L. perolatii* (NCBI: WP_100713649.1), *L. broomii* (NCBI: WP_020987394.1), *L. inadai* (NCBI: WP_068872775.1), and four saprophytic species of the clade S: *L. biflexa* (UP: B0SJW7), *L. bourretii* (NCBI: WP_135748451.1), *L. jelokensis* (NCBI: WP_135751084.1), and *L. ryugenii* (UP: A0A2P2DVA2).

### 2.5 Prediction of leptolysin stability in pathogenic, intermediate, and saprophytic leptospires

Multi-species alignment of *Leptospira* leptolysin sequences disclosed non-conserved residues neighboring zinc, calcium, and arginine binding sites. The server Duet (http://biosig.unimelb.edu.au/duet/stability) was used to predict the effects of amino acid variations - found in pathogenic, intermediate, and saprophytic leptospires - on leptolysin stability.

### 2.6 Cloning of the gene coding for *L. interrogans* leptolysin

The sequence corresponding to full-length leptolysin but excluding the N-terminal signal peptide (amino acids 1 – 22) was amplified by PCR from genomic DNA of *L. interrogans* serovar Copenhageni strain 10A using the following primers: F – 5′ GGATCCAAAGGCAAAGACGATAATTCTAAAAAC 3′ and R - 5′ CCATGGTTAATATACAAGAGGGTGAGAACGG 3’. The amplicon was cloned into the pGEM-T Easy vector (Promega Corp., Madison, WI), and transformed into *Escherichia coli* DH5α. Following digestion with BamHI and NcoI, the fragment was subcloned into the pAE vector for the expression of the recombinant protein with an N-terminal 6×His tag (Ramos et al., 2004). Constructs were verified by DNA sequencing with vector-specific primers.

### 2.7 Expression and purification of *L. interrogans* leptolysin

*Escherichia coli*, strain BL21(DE3), was transformed with the pAE-leptolysin plasmid and inoculated into 1 L of the rich medium two-fold HKSII (Jensen and Carlsen, 1990) containing ampicilin (100 μg/mL), and grown at 200 rpm at 37°C. When OD600 nm reached 3.0, 0.5 mM of isopropyl-b-D-thiogalactoside (IPTG) was added to the cultures which were maintained under agitation at 150 rpm for further 16 h at 30°C. The cultures were centrifuged at 12,000 x *g* for 15 min at 4°C, and the pellet was resuspended in lysis buffer (0.1 M Tris HCl, pH 8.0, 5 mM EDTA, containing 50 µg/mL lysozyme and 0.1% sodium deoxycholate) and lysed by sonication. The leptolysin-Inclusion Bodies (IBs) were washed twice in wash buffer (0.1 M Tris HCl, pH 8.0, 5 mM EDTA and 0.1% sodium deoxycholate), centrifuged and resuspended in a buffer containing 0.05 M Tris HCl pH 8.0 and 1 mM EDTA, then centrifuged and resuspended in the same buffer. Aliquots of the suspension were kept in -20°C (Chura-Chambi et al., 2013). Solubilization of leptolysin-IBs at high hydrostatic pressure was achieved by diluting 1 mg of IBs in a buffer containing 50 mM CAPS pH 11.0, 1 mM EDTA and 0.4 M arginine. The suspension was placed into plastic bags that were sealed, placed inside another plastic bag, which was vacuum sealed, placed into a pressure vessel (R4-6-40, High Pressure Equipment, USA) and pressurized at 2.4 kbar for 90 min using oil as a transmission fluid in a suitable high-pressure pump (PS-50, High Pressure Equipment, USA) at 20°C. After decompression, the sample was centrifuged at 12,000 x *g* for 15 min and dialyzed against 25 mM sodium phosphate buffer at pH 8.0 and 4°C. Soluble leptolysin was centrifuged again and further purified by size exclusion chromatography. The protein was applied to Superdex 200 10/300 column (GE Healthcare Life Sciences) coupled to an ÄKTA Purifier (GE Healthcare) system. The buffer used for elution was 25 mM sodium phosphate pH 8.0 using a flow rate of 1 mL/min. Protein concentration was estimated by the bicinchoninic acid assay (Pierce™ BCA Protein Assay Kit).

### 2.8 Antiserum against recombinant *L. interrogans* leptolysin

Antiserum against recombinant leptolysin was obtained in accordance with the ethical principles of the Committee on Ethics of Instituto Butantan (protocol # 9452010316, approved in the meeting of 03/16/2016) which is in agreement with Law 11.794, of October 8 2008, Decree 6899, of July 15, 2009, with the rules issued by the National Council for Control of Animal Experimentation (CONCEA).

A male healthy New Zealand rabbit of approximately 60 days old was provided by the Central Animal Facilities of Instituto Butantan. The animal was immunized intramuscularly with 75 µg of recombinant protein and 2.0 mg of aluminum hydroxide as adjuvant (total volume 500 µL). Two subsequent booster injections were given at 15 day intervals. The animal was bled from the marginal ear vein prior to immunizations (pre-immune bleeding/serum control), and two weeks after the third dose total bleeding by cardiac puncture was performed under deep terminal anaesthesia. The blood was centrifuged for 5 min at 3000 x *g* and the serum was stored at –20°C. Antibody titers were determined by enzyme-linked immunosorbent assay (ELISA). Wells of a 96-well plate were coated with 0.5 µg of recombinant leptolysin diluted in 100 µL of 0.1 M sodium bicarbonate solution pH 9.6, and incubated overnight at 4°C. The plates were washed 3 times with PBS + Tween-20 (PBST; 0.05% Tween-20), and then blocked with 10% non-fat powdered milk overnight at 4°C. The plates were washed with PBST and incubated with 100 µL rabbit antiserum against leptolysin serially diluted from 1: 50 to 1:102,000 for 1 h at 37°C. Non-immune serum was used as a negative control. Following 3 washes with PBST, plates were incubated with 100 µL diluted horseradish peroxidase (HRP)-conjugated goat anti-rabbit IgG (1:5,000 dilution; Sigma-Aldrich, St. Louis, MO, USA) for 1 h at 37°C. Substrate reaction was performed with o-phenyldiamine dihydrochloride (Pierce, Thermo Fisher Scientific Inc., Rockford, USA) and absorbance was measured at 492 nm using a Multiskan EX microplate reader (Thermo Fisher Scientific Inc., Waltham, USA).

### 2.9 *Leptospira* strains and growth conditions

The virulent strains *Leptospira interrogans* serovar Copenhageni strain Fiocruz L1-130 (L1-130), *Leptospira interrogans* serovar Kennewicki strain Fromm, and *Leptospira interrogans* serovar Manilae strain L495, the culture-attenuated *Leptospira interrogans* serovar Copenhageni strain 10A and *Leptospira kirschneri* serovar Cynopteri strain 3522C, and the saprophytes *Leptospira biflexa* serovar Andamana strain CH11 and *Leptospira biflexa* serovar Patoc strain Patoc I were provided by the Laboratory of Bacterial Zoonosis, School of Veterinary Medicine and Animal Sciences, University of São Paulo, Brazil. Virulence of the strains L1-130, Fromm and L495 was maintained by successive inoculations in hamsters. Strains were cultured, and supernatants were collected and stored as previously described (Oliveira et al., 2021).

### 2.10 Western blot

Total proteins from supernatants (3 μg) were subjected to 12% SDS-PAGE and transferred to a nitrocellulose membrane. After blocking with 10% skimmed milk in PBST, leptolysin was detected with the antiserum produced in rabbit at a 1:5,000 dilution followed by peroxidase-conjugated anti-rabbit IgG (1:10,000) incubation. Positive signals were detected by enhanced chemiluminescence (West Pico, Pierce) using Alliance HD6, an Uvitec chemiluminescence Documentation System (Uvitec, Cambridge, UK).

### 2.11 Fluorescent resonance energy transfer (FRET) peptides and reagents

The FRET substrates Abz-GLARSNL-EDDnp and Abz-GLQRALEI-EDDnp were purchased from GenOne Biotecnologies (Rio de Janeiro, Brazil). The substrates Abz-GGLFLRR-EDDnp, Abz- Abz-FLRRV-EDDnp, and Abz-RPPGFSPFRQ-EDDnp were kindly provided by Prof. Luiz Juliano Neto, from the Department of Biophysics of UNIFESP-EPM, São Paulo, Brazil. The peptidase inhibitors ethylene diamine tetraacetic acid (EDTA), phenylmethylsulfonyl fluoride (PMSF), and 1,10-phenantroline were purchased from Sigma-Aldrich (St Louis, MO, USA). E-64 was purchased from Tocris Bioscience (Minneapolis, MN, USA).

### 2.12 Effect of pH, temperature, cation, and site-directed inhibitors influence on *L. interrogans* leptolysin activity

Leptolysin activity parameters were analyzed using 0.5 µg of protein, 5 µM of the FRET substrate Abz-GLARSNL-EDDnp and the assay buffer. All assays were performed at the final volume of 100 µL and monitored on a fluorimeter Hidex Sense 425-301 (Turku, Finland) adjusted for excitation and emission readings at 320 and 420 nm, respectively. The temperature remained constant at 37°C (except in the temperature assay) and the measurements of peptidase activity were made for 15 or 30 min continuously (one read per minute). The fluorometric assays were analyzed using GraphPad Prism 5 software (San Diego, CA, USA) and the specific activities were determined. All fluorometric measurements were made in triplicate, and the results are shown as the mean with SD. To determine the influence of pH on leptolysin activity, buffers were prepared according to Stoll and Blanchard (1990), each one at the final concentration of 50 mM: sodium phosphate (pH 5.0 – 7.5), borate buffer (pH 8.0 – 9.0) and borate buffer-NaOH (pH 9.5 -10.0).

The activity of leptolysin in 50 mM borate buffer pH 8.0 in the presence of mono and divalent cations as their chlorides (K^+^, Na^+^, Li^+^, Ca^2+^ and Mg^2+^) at the final concentration of 50 mM was then analysed.

The effect of temperature on leptolysin activity was evaluated within the temperature range of 25 to 42°C, with addition of the substrate 15 min after incubation at each temperature in 50 mM borate buffer, 50 mM NaCl pH 8.0.

Class-specific inhibitors effect on leptolysin activity was evaluated using 50 mM EDTA, 2 mM 1-10-phenantroline, 2 mM PMSF and 10 µM E-64. PMSF and 1,10-phenantroline were incubated with leptolysin for 30 min before addition of the substrate. The same volume of ethanol was used as a control condition.

All data are presented as mean ± SD in triplicate. The results on the influence of salts and site-directed inhibitors on the proteolytic activity of leptolysin were analyzed using one-way analysis of variance (ANOVA) for between-groups comparisons followed by a Tukey’s *post-hoc* test for multiple comparisons. Values of *p* < 0.05 were considered to be statistically significant. The analyses were performed using GraphPad Prism 6.0 software (GraphPad Software, Inc., La Jolla, CA).

### 2.13 Determination of hydrolysis points and specific activity over FRET substrates

The cleavage points produced by *L. interrogans* leptolysin on the fluorescent substrates were determined by mass spectrometry analysis. Hydrolysis of the substrates (5 µM) in 50 mM borate, 50 mM NaCl pH 8.0 (final volume 100 μL) and 0.5 µg of leptolysin was monitored on Hydex Sense 425-301 fluorimeter (Turku, Finland), as described above, and the cleavage points were determined in a MALDI-TOF mass spectrometer. The scissile bonds were deduced from the sequences of the substrate fragments, and these analyses were performed on Aimal Performance equipment (Shimadzu Co, Japan) in the linear positive mode. One microliter of each sample was co-crystallized with α-cyano-4-hydroxycinnamic acid (acetonitrile/water saturated solution/0.1% TFA – matrix), deposited on the sampler and dried at room temperature. The samples were analyzed and the spectra were obtained using the linear positive mode.

The specific activities were determined by the incubation of leptolysin (0.23 – 1.16 µg) with the FRET substrates (5 µM), as described above, and results were attained using the GraphPad Prism 5 software (San Diego, CA, USA), and expressed by µM of substrate consumed by µg of leptolysin per minute (µM/µg/min).

### 2.14 Degradation of ECM molecules

Recombinant *L. interrogans* leptolysin (0.1 μg) was incubated with 0.5 μg of proteoglycans mimecan (# 2949-MC), lumican (# 2846-LU), biglycan (# 2667-CM) and decorin (# 143-DE) (R & D Systems, Inc., Minneapolis, USA), and with 5 µg of plasma fibronectin (Sigma-Aldrich, St. Louis, MO, USA) for up to 4 h in 50 mM Tris HCl, 200 mM NaCl, 10 mM CaCl_2_ and 0.05% CHAPS, pH 7.4, at 37°C. Dose-dependent degradation was assessed by incubating each substrate with increasing amounts of leptolysin (0.025 – 0.2 μg). The assays were also performed in the presence of metalloprotease inhibitors by preincubating the recombinant protease with 5 mmol/L 1,10-phenanthroline or 10 mM EDTA for 30 min before the addition of each substrate. In control samples, each substrate was incubated for 4 h in the assay buffer under identical conditions. Reactions were submitted to 12% - SDS-PAGE followed by electroblotting onto nitrocellulose membranes. Degradation products were detected using goat anti-human IgG primary antobodies (R & D Systems, Inc., Minneapolis, USA): anti-mimecan (# AF2949, 1:3,750), anti-lumican (# AF2846, 1:7,500), anti-biglycan (# AF2667, 1:3,750) and anti-decorin (# AF143, 1:3,750), followed by peroxidase-conjugated rabbit anti-goat antibodies (Sigma-Aldrich, St. Louis, MO, USA). Positive signals were detected b chemiluminescence (West Pico, Pierce) using Alliance HD6, an Uvitec chemiluminescence Documentation System (Uvitec, Cambridge, UK). Fibronectin degradation was assessed by SDS-PAGE and subsequent silver staining (rapid silver nitrate protocol) (Rabilloud et al., 1994).

### 2.15 Effect of *L. interrogans* leptolysin on HK-2 cells

HK-2 cells, an immortalized proximal tubule epithelial cell line from normal adult human kidney, were cultured in Dulbbelco’s Modified Eagle Medium/Ham’s F12 supplemented with 5% fetal bovine serum, 2 mM glutamine, 20 mM HEPES, 0.4 µg/mL hydrocortisone, 5 µg/mL insulin, 5 µg/mL transferrin and 5 µg/mL sodium selenite (Tian et al., 2006). Cells were detached by TryplE express (Gibco, EUA) according to manufacturer´s instructions, and were counted using a Neubauer chamber. They were seeded on 96-well plates at 2.5 x 10^5^ cells/well, and were maintained at 37°C and 5% CO_2_ until they reached 60-70% confluence. Culture medium without fetal bovine serum was added to the cells 24 h before treatment with leptolysin to synchronize cell cycle. Cells were incubated with 0.1 µg, 0.5 µg or 1 µg of leptolysin, or with 5 µg of *L. interrogans* Manilae L495 supernatant for 24 h. Cells were also treated with leptolysin (0.5 µg) or Manilae L495 supernatant (5 µg) preincubated for 30 min at 37°C with leptolysin antiserum (1: 100). Negative controls included cells with culture medium without fetal bovine serum and cells incubated with buffer (50 mM Tris HCl, 200 mM NaCl, 10 mM CaCl_2_ and 0.05% CHAPS, pH 7.4). Cells were visualized by light microscopy.

### 2.16 Immunofluorescence

HK-2 cells were seeded on 6-well plates containing 18 mm glass coverslips (Knittel, #100018) at a concentration of 1x10^5^ cells/mL, and incubated in 5% CO_2_ at 37°C for 72 h to reach a confluence of 60-70%. The culture medium was then replaced by medium without fetal bovine serum and after 24 h the cells were treated with 1 µg of recombinant leptolysin for 24 h. The culture supernatant was then removed, and cells were fixed with 2% formalin for 15 min and washed with PBS three times. They were then permeabilized with 0.1% Triton X-100 diluted in PBS for 4 min. Cells were washed again as described, and blocked with PBS -BSA 2% for 30 min before the addition of anti-human fibronectin produced in rabbit (Sigma-Aldrich, St. Louis, MO, USA, # F3648) (1:7,500) or anti-human zonula occludens produced in rabbit (Thermo Fisher Scientific, Waltham, MA, USA, # 61-7300) (1:500) diluted in PBS-BSA 2%. Incubation proceeded for 45 min and after three washes, the cells were incubated with FITC-conjugated anti-rabbit (Sigma-Aldrich, St. Louis, MO, USA, # F0382) or FITC-conjugated anti-mouse (Thermo Fisher Scientific, Waltham, MA, USA) secondary antibodies diluted in PBS-BSA 2% containing propidium iodide at a dilution of 1: 1,000 for 1 h. Cells were washed with PBS three times. A drop of Citifluor Mounting Solution (Electron Microscopy Sciences, Hatfield, PA, USA, # 17976-25) was placed on a microscope slide and coverslips were placed with the face containing the cells in contact with the mounting solution and sealed with nail polish. Analysis and image records were performed using a confocal laser scanning microscope (TCS SP8, Leica, Germany). The Leica software LAS X was used for image acquisition, and the objective lens used was the HC PL APO CS 63 × 1.20 W.

### 2.17 Injection of leptolysin in the mouse skin

This protocol was performed in accordance with the ethical principles of the Committee on Ethics of Instituto Butantan (protocol # 1033050321, approved in the meeting of 03/30/2021) which is in agreement with Law 11.794, of October 8 2008, Decree 6899, of July 15, 2009, with the rules issued by the National Council for Control of Animal Experimentation (CONCEA).

Male Swiss mice (n = 2 per group) weighing 25 - 30 g were injected intradermally on the dorsal region with buffer (50 mM Tris HCl, 200 mM NaCl, 10 mM CaCl_2_ and 0.05% CHAPS, pH 7.4), buffer containing 2, 4 or 6 μg of leptolysin, or buffer containing 4 μg of leptolysin preincubated for 30 min with 5 mmol/L 1,10-phenanthroline. After 4 h mice were euthanized, and the dorsal skin was removed and homogenized with a tissue homogenizer (Omnimixer G20, Omni TH^®^, Omni International) using lysis buffer (50 mM HEPES, 200 mM NaCl, 2% CHAPS, pH 7.5) containing the cOmplete Protease Inhibitor Cocktail (Roche, Mannheim, Germany) with 4,000 rpm cycles for 30 s (3 times) in ice bath. Samples were centrifuged at 14,000 × *g*, for 10 min at 4°C to remove debris and the supernatants were stored for analysis (Asega et al., 2020). Skin proteins from control and leptolysin-treated animals (4 μg) were submitted to 10% SDS-PAGE (for fibronectin detection) or 12% SDS-PAGE (for β-actin detection) followed by Western blot analysis as previously described using anti-fibronectin produced in rabbit (1: 5,000) and anti β-actin produced in mouse (1: 100,000) (# 3648 and # A5441, respectively, Sigma-Aldrich, St. Louis, MO, USA), and peroxidase-conjugated anti-rabbit IgG (1:5,000) or anti-mouse (1: 10,000) incubation. Positive signals were detected by enhanced chemiluminescence (West Pico, Pierce) using Alliance HD6, an Uvitec chemiluminescence Documentation System (Uvitec, Cambridge, UK).

## 3 Results

### 3.1 Comparative analysis of leptolysin

**Figure 1:**
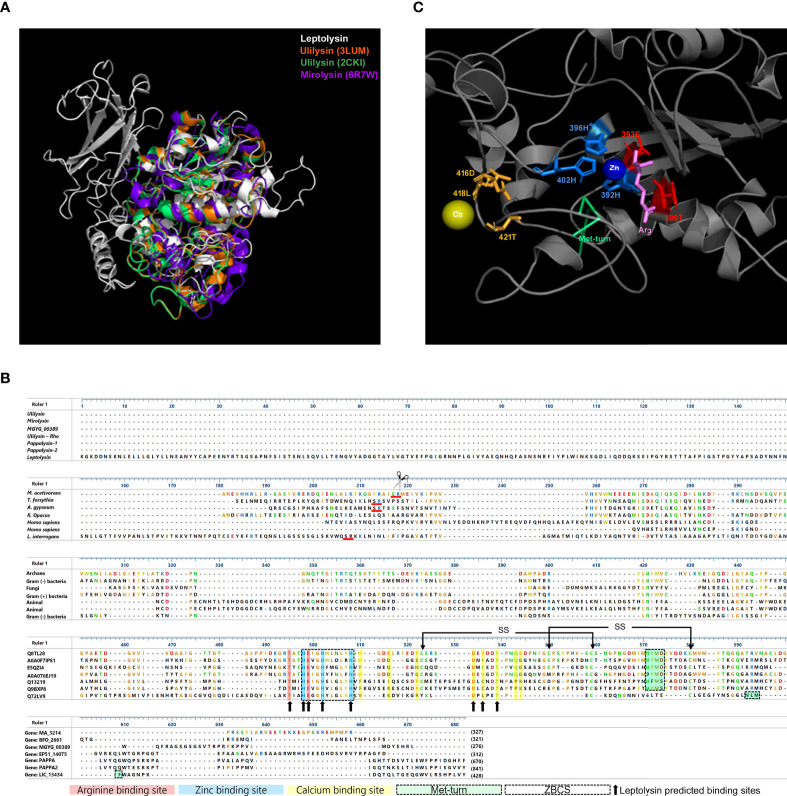
Comparative analysis of short pappalysins. **(A)** 3D structure of leptolysin structurally aligned to ulilysin 3LUM (orange) and 2CKI (green) and mirolysin (purple); **(B)** M43 metalloproteases alignment: *L. interrogans* leptolysin, *M. acetivorans* ulilysin, *T. forsythia* mirolysin, *R. Opacus* ulilysin, *A. gypseum* extracellular metalloprotease MGYG_00389 and *H. sapiens* pappalysin-1 and pappalysin-2 were aligned by Muscle. The zinc-binding consensus sequence (ZBCS) is boxed. Ulilysin arginine (red), zinc (blue), and calcium (yellow) -binding sites are highlighted, while corresponding leptolysin residues within those binding sites are indicated by black arrows. Met-turn structures are boxed and highlighted in green. Red lines indicate Serine-Argine residues, site of autolytic cleavage of ulilysin and mirolysin. Ulilysin disulfide bonds (SS) are indicated by arrows above cysteines. **(C)** *L. interrogans* leptolysin predicted binding sites: calcium-binding sites are indicated by gold sticks, zinc-binding sites are indicated by blue sticks and arginine binding sites are highlighted in red. The Met-turn is shown in green. Calcium (Ca) and zinc (Zn) ligands are represented by yellow and blue spheres, respectively, while arginine (Arg) is indicated by pink sticks.

A 3D structural model of *L. interrogans* leptolysin was generated by comparative modeling using the protein ulilysin from *Methanosarcina acetivorans*, which harbors a pappalysin-1 domain. Leptolysin model was then aligned to *Methanosarcina acetivorans* ulilysin (PDB: 3LUM and 2CKI) and *Tannerella forsythia* mature mirolysin (6R7W). Superposition of the three crystallographic structures discloses similarities in protein folding regarding their C-terminal regions, which harbor the catalytic domain (**Figure 1A**). Sequence alignment of those three M-43 metalloproteases reveals that ulilysin and mirolysin present a site of autolytic cleavage for their activation, underlined in red (scissors indicate cleavage of the Ser-Arg peptide bond that allows removal of the N-terminal pro-domain; **Figure 1B**). Interestingly, leptolysin also presents a Ser-Arg site at position 230, but it seems that this protease does not undergo autolytic cleavage, as discussed later.

Prediction of leptolysin calcium, zinc, and arginine -binding sites were performed using the Coach server. Residues 416D, 418L and 421T were predicted as calcium-binding sites, while histidines (H) at positions 392, 396 and 402 were predicted as zinc-binding sites, and residues 389T and 393E as arginine binding sites (**Figure 1C**). Alignment of leptolysin with M-43B metalloproteases from Archaea (*M. acetivorans*), Gram-negative and Gram-positive bacteria (*T. forsythia*, *R. opacus*, respectively), fungi (*A. gypseum*) and *Homo sapiens* discloses the highly conserved extended zinc-binding consensus sequence (ZBCS), HEXXHXXGXXH/D, and corroborates the prediction of leptolysin residues 416D and 421T as calcium-binding sites, since they are conserved and already characterized in the other proteins. Moreover, our data suggest that the amino acids _450-_NLMF_-453_ correspond to the leptolysin methionine-containing 1,4-β-turn (Met-turn). Although not perfectly aligned with the Met-turn of compared proteins, its location below the zinc binding site on the 3D structure supports its identification as a Met-turn structure (**Figure 1B**). The cysteine residues involved in disulfide bridges formation in mirolysin and ulilysin are not fully conserved in leptolysin.

### 3.2 Leptolysin catalytic domain is highly conserved among pathogenic *Leptospira* species

**Figure 2:**
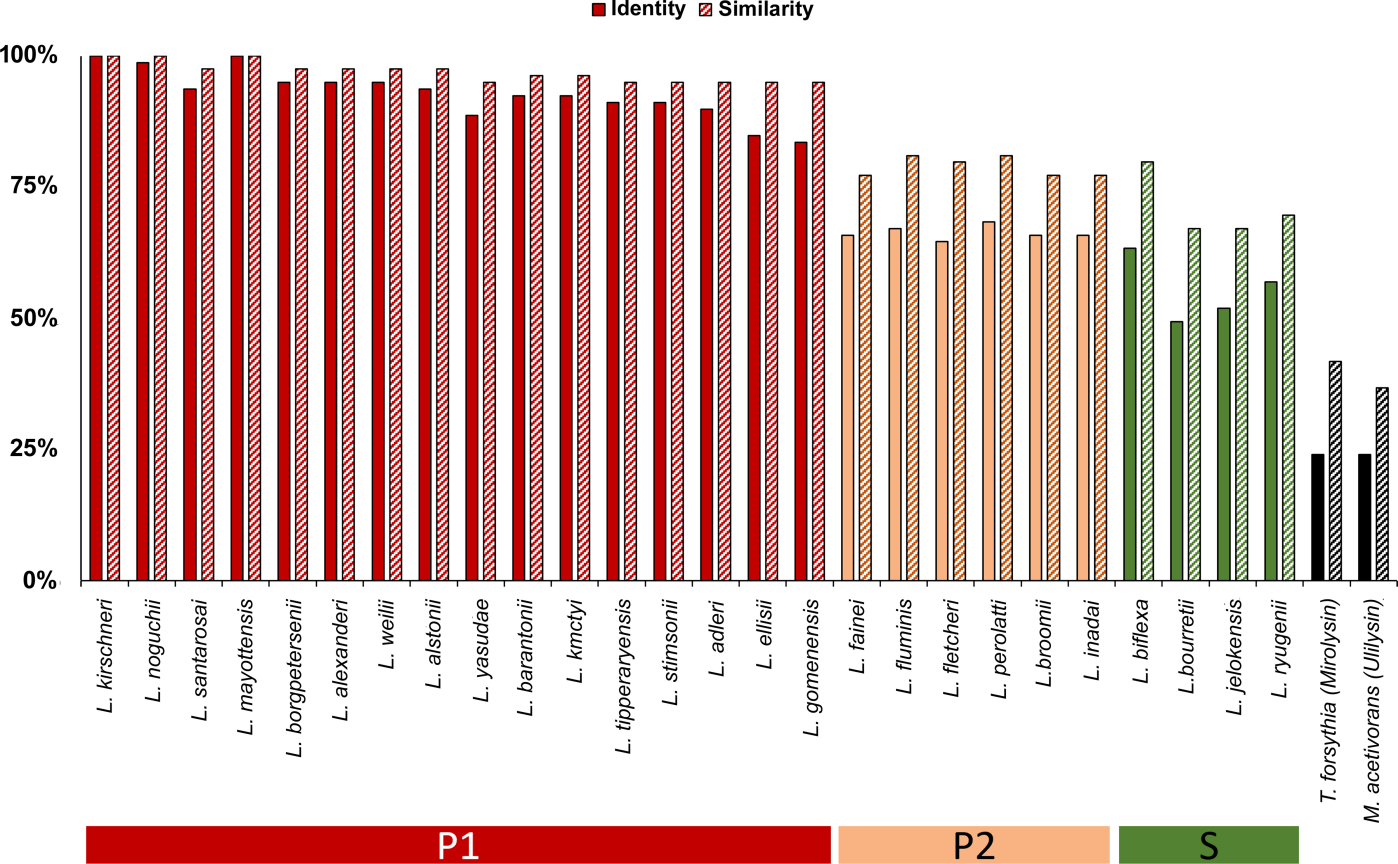
Conservation of leptolysin catalytic domain among *Leptospira* spp. Multiple sequence alignment of leptolysin catalytic domain was performed with MUSCLE software (Edgar, 2004), and included 17 *Leptospira* species of the subclade P1, 6 species of the subclade P2, 3 species of the subclade S1, and 1 species of the subclade S2. Mirolysin and ulilysin catalytic domains were also included. Leptolysin sequence similarities of all species are presented as a percentage value relative to the protein sequence of *L. interrogans* serovar Copenhageni L1-130 (WP_000914951.1).

Leptolysin catalytic domain sequence conservation was assessed in 17 pathogenic species of the subclade P1, 6 species of the subclade P2, and 4 saprophytic species (clade S). Sequence conservation was higher among pathogenic leptospires (84% – 100% identity, 95% – 100% similarity). As expected, leptolysin catalytic domain was less conserved among species of the subclade P2 (65% - 68% and 77% - 81% of identity and similarity, respectively) and among saprophytic leptospires (49% - 63% identity and 67% - 80% similarity). Mirolysin and ulilysin catalytic domains share only 25% identity, and 42% and 37% similarity, respectively, with leptolysin catalytic domain (**Figure 2**).

**Figure 3:**
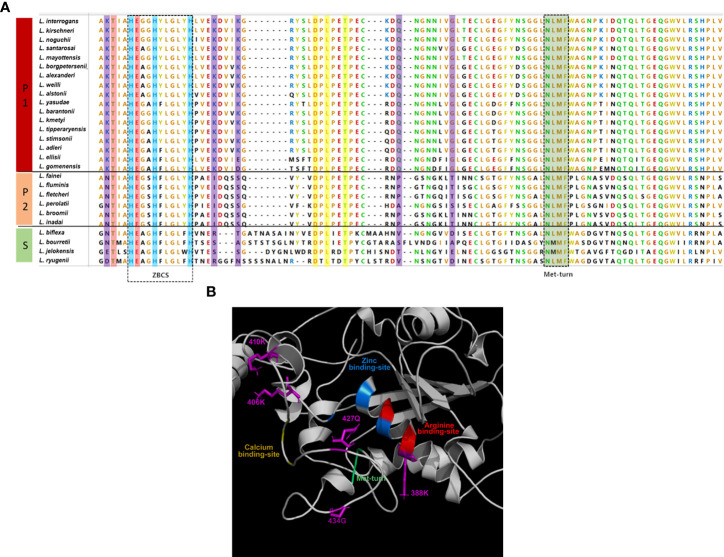
Comparative analysis of leptolysin catalytic domain. **(A)** Pathogenic (P1), intermediate (P2) and saprophytic (S) leptolysins (amino acids 387 - 480) were aligned by Muscle. Zinc, calcium, and arginine binding sites are colored in blue, yellow, and red, while the Met-turn is colored in green; variable residues are colored in purple; **(B)** Zinc, calcium, and arginine -binding sites, Met-turn and variants found in P2 and S leptolysins at positions K388, K406, K410, Q427 and G434 (purple) are indicated.

Multiple sequence alignment shows that the extended ZBCS, the Met-turn, and the calcium, zinc, and arginine -binding sites of leptolysin are well conserved among species of all *Leptospira* subclades (**Figure 3A**). However, non-conserved residues in the catalytic domain of leptolysins from subclades P2 and S are also observed: lysines (K) at positions 388, 406, and 410; glutamine (Q) at position 427, and glycine (G) at position 434 are only conserved among species of the subclade P1 (**Figure 3A**).

Considering the proximity of these particular residues to the calcium, zinc, and arginine -binding sites and to the Met-turn (**Figure 3B**), we investigated if those amino acid alterations would impact protein stability. Interestingly, fifteen out of seventeen variants found in P2 and S leptolysins at positions K^388^, K^406^, K^410^, Q^427^ and G^434^, were predicted as destabilizing, resulting in negative folding free energy (**Table 1**). Conversely, K410E and K410D variants found in P1 leptolysins were predicted as stabilizing, which strongly suggests maintenance of the catalytic site structure.

**Table 1:** Effects of residue variations on leptolysin catalytic site stability.

Position	P1 residue	Substitution	Found in	ΔΔG (kcal/mol)	Effect
388	K	N	P2; S	-1.402	**Destabilizing**
		E	S	-1.477	**Destabilizing**
		D	S	-0.797	**Destabilizing**
406	K	I	P2	0.599	Stabilizing
		R	S	-0.272	**Destabilizing**
		S	S	-1.51	**Destabilizing**
410	K	S	P2; S	-0.05	**Destabilizing**
		E	P1	0.494	Stabilizing
		D	P1	0.475	Stabilizing
427	Q	P	P2	-0.243	**Destabilizing**
		A	P2	-0.401	**Destabilizing**
		V	S	0.286	Stabilizing
		S	S	-0.907	**Destabilizing**
		T	S	-0.449	**Destabilizing**
434	G	T	P2	-1.205	**Destabilizing**
		S	P2	-1.397	**Destabilizing**
		D	S	-0.815	**Destabilizing**
		A	S	-0.811	**Destabilizing**
		E	S	-0.79	**Destabilizing**

* ΔΔG indicates the predicted change in folding free energy with the modified residue (kcal/mol). A negative value corresponds to a residue predicted to destabilize the protein structure (bold), while a positive value corresponds to a residue predicted to have a stabilizing effect.

### 3.3 Recombinant production and detection of leptolysin in *Leptospira* culture supernatants

**Figure 4:**
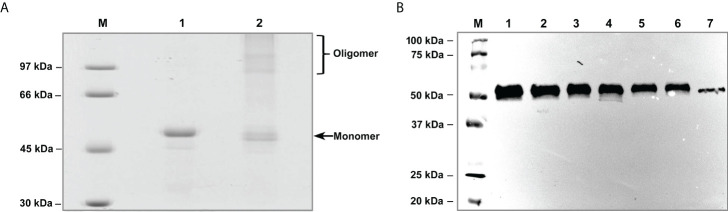
Purification of recombinant *L. interrogans* leptolysin and detection of the native protease in *Leptospira* culture supernatants. **(A)** Purified recombinant leptolysin was analysed under reducing (1) and nonreducing (2) conditions. Proteins were separated by SDS/12% PAGE and stained with Coomassie Brilliant Blue. M: molecular mass marker. **(B)** Production of leptolysin by (1) *Leptospira interrogans* serovar Copenhageni strain Fiocruz L1-130, (2) *Leptospira interrogans* serovar Kennewicki strain Fromm, (3) *Leptospira interrogans* serovar Copenhageni strain 10A, (4) *Leptospira kirschneri* serovar Cynopteri strain 3522C, and the saprophytes (5) *Leptospira biflexa* serovar Andamana strain CH11 and (6) *Leptospira biflexa* serovar Patoc strain Patoc I; (7) Recombinant leptolysin. Proteins from *Leptospira* supernatants were separated by SDS/12% PAGE, transferred to nitrocellulose membranes, and probed with leptolysin antiserum produced in rabbit.

Production of enzymatically active recombinant *L. interrogans* leptolysin was achieved by refolding the protein expressed as inclusion bodies in *Escherichia coli* by the application of high hydrostatic pressure. After purification, the protein appears as a single band of approximately 50 kDa on the SDS-PAGE gel (**Figure 4A**, lane 1). Although cysteine residues are not fully conserved in leptolysin, formation of intra and intermolecular disulfide bonds cannot be discarded, since the protein migrates slightly faster as a monomer but also forms homooligomers under nonreducing conditions (**Figure 4A**, lane 2).

As leptolysin has a signal peptide for secretion, protein production by *Leptospira* serovars belonging to different species was assessed by immunoblotting using culture supernatants. Both pathogenic and saprophytic leptospires produce leptolysin, visualized as a ~50 kD protein band (**Figure 4B**).

### 3.4 Characterization of *L. interrogans* leptolysin activity by FRET

#### 3.4.1 Biochemical characterization: effect of pH, salt concentration, temperature and site-direct inhibitors

**Figure 5:**
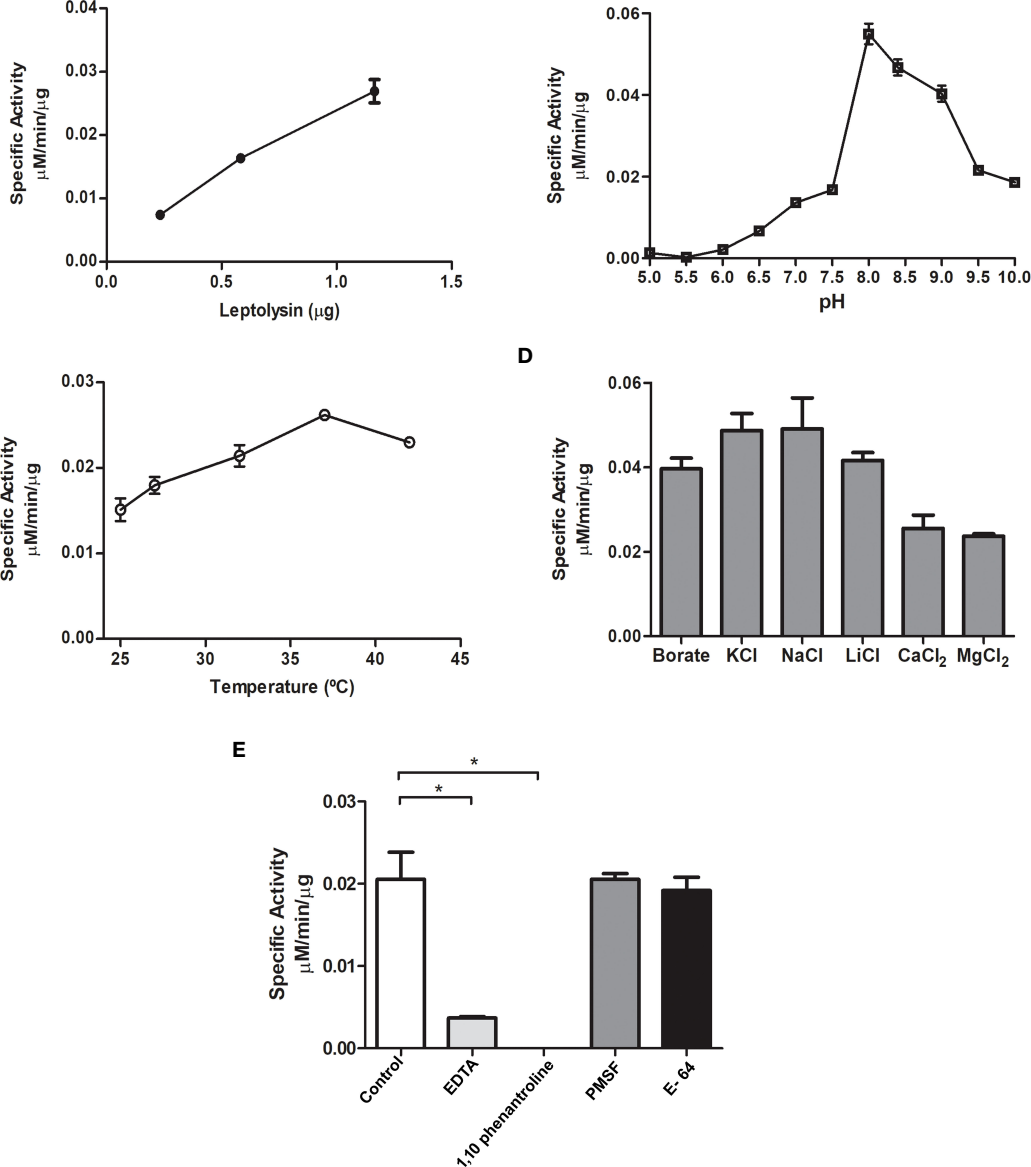
Initial biochemical characterization of *L. interrogans* leptolysin using the Abz-GLARSNL-EDDnp peptide (5 μM) as substrate. **(A)** Dose-response curve using three different leptolysin amounts (0.3 µg, 0.6 µg and 1.2 µg); **(B)** Evaluation of optimum pH (pH 5 to pH 10); **(C)** Thermo-stability study between 25 °C and 42 °C; **(D)** Influence of monovalent and divalent cations on leptolysin activity; **(E)** Effect of site-directed inhibitors on leptolysin catalytic activity. All assays were made in triplicate and data are presented as mean ± SD The results on the influence of salts **(D)** and site-directed inhibitors **(E)** on the proteolytic activity of leptolysin were analyzed using one-way analysis of variance (ANOVA) for between-groups comparisons followed by a Tukey’s post-hoc test for multiple comparisons (* p < 0.05).

Enzymatic activity of *L. interrogans* leptolysin was initially assessed using synthetic FRET peptides. One of those substrates, Abz-GLARSNL-EDDnp, was efficiently hydrolyzed by leptolysin, and was then selected for further biochemical characterization. Kinetic studies were performed to evaluate the effects of pH, temperature, and salts on protease activity. As shown in **Figure 5**, increasing amounts of leptolysin intensified the fluorescent signal in a dose-dependent manner (A). The enzyme exhibited maximal activity at pH 8.0 (B) and 37°C (C), and hydrolytic activity was observed in the presence of different salts, but no statistically significant difference was observed between them (D). The metalloprotease proteolytic action (control) was strongly inhibited by both EDTA and 1,10-phenanthroline (*p* < 0.05), but not by E-64 or PMSF (E).

#### 3.4.2 Analysis of the cleavage sites and specific activities

The cleavage point produced by *L. interrogans* leptolysin on the fluorescent substrate Abz-GLARSNL-EDDnp, used for biochemical characterization, was determined. Leptolysin produced a single Arg-Ser cleavage point. The scission bonds produced by the protease were also determined for the substrates Abz-GGFLRR-EDDnp, Abz-FLRRV-EDDnp, Abz-RPPGFSPFRQ-EDDnp and Abz-GLQRALEI-EDDnp (**Table 2**), which allowed a preliminary analysis of its primary specificity. Abz-RPPGFSPFRQ-EDDnp was the most susceptible substrate for leptolysin, presenting a specific activity of 0.45 µM/µg/min, followed by Abz-FLRRV-EDDnp (0.3 µM/µg/min) and Abz-GLASRRV-EDDnp (0.19 µM/µg/min). According to the results presented in **Table 2**, and the data obtained by the IceLogo software (**Supplementary Figure 1**), the preference for arginine in the P1 position was significant for leptolysin, as all substrates tested were cleaved after this particular residue.

**Table 2:** Specific activities and cleavage points produced by *L. interrogans* leptolysin on FRET substrates.

Specific activity		Substrate										
(µM/µg/min)			P5	P4	P3	P2	P1	P1’	P2’	P3’	P4’	
**0.19 ± 0.01**	Abz		–	G	L	A	R	S	N	L	–	EDDnp
**0.02 ± 0.01**	Abz		–	G	L	Q	R	A	L	E	I	EDDnp
**0.30 ± 0.05**	Abz		–	–	F	L	R	R	V	–	–	EDDnp
**0.09 ± 0.01**	Abz		G	G	F	L	R	R	–	–	–	EDDnp
**0.45 ± 0.01**	Abz	RPPG	F	S	P	F	R	Q	–	–	–	EDDnp

The peptides were incubated with *L. interrogans* leptolysin and the specific activities were determined in a fluorimeter. The hydrolysis products were analyzed by mass spectrometry to determine the cleavage sites.

Despite the marked preference for arginine residues in the P1 position, differences regarding specific activities for the hydrolysis of Abz-GGFLRRV-EDDnp (0.09 µM/µg/min) and Abz-FLRRV-EDDnp (0.3 µM/µg/min) indicates that leptolysin has an extended binding site for its association with substrates, since the addition of two glycine residues at the N-terminus of the substrate reduced the specific activity by more than three times. However, further studies using a larger number of substrates will be necessary to confirm this issue.

### 3.5 Degradation of proteoglycans and plasma fibronectin by *L. interrogans* leptolysin

Since leptolysin presented proteolytic activity on synthetic FRET peptides, degradation of small leucine-rich proteoglycans (SLRP) was assessed. Ubiquitously distributed in ECM, connective tissues, and on the surface of diverse cell types, SLRP were found to be targeted by *L. interrogans* proteases released in the ECM (da Silva et al., 2018).

**Figure 6:**
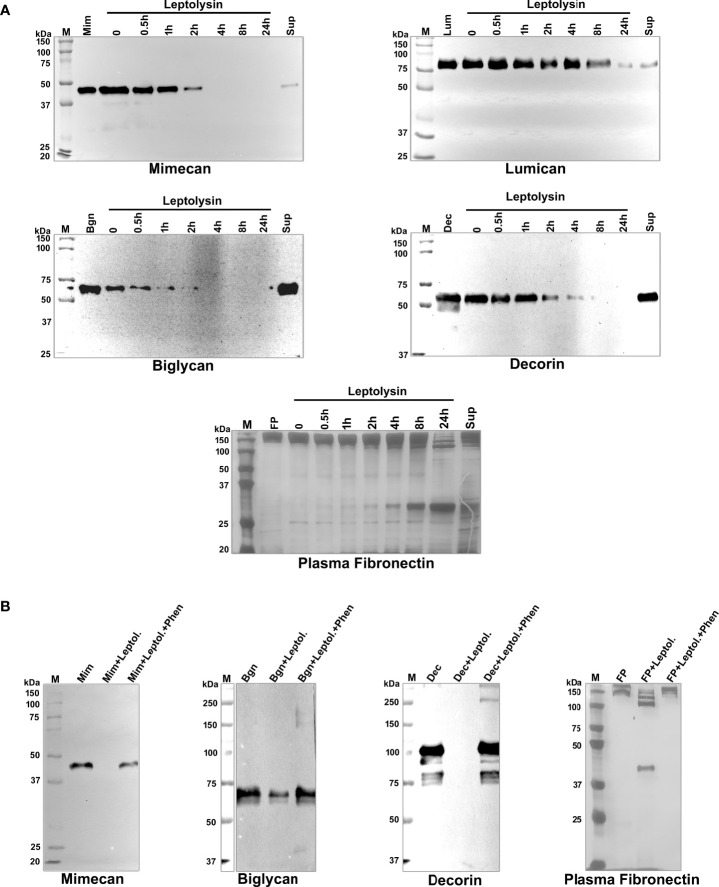
Proteolytic activity of *L. interrogans* leptolysin on proteoglycans and plasma fibronectin. **(A)** Degradation of substrates: mimecan, lumican, biglycan, decorin (0.5 μg), and plasma fibronectin (5 μg) were incubated with leptolysin (0.1 µg) at 37°C for the indicated time points. Cleavage products were subjected to SDS- polyacrylamide gel under reducing conditions, transferred to nitrocellulose membranes, and probed with specific antibodies, or the gel was silver stained (FP). **(B)** Inhibition of proteolytic activity: before the addition of each substrate, leptolysin (0.1 µg) was incubated with 5 mmol/L 1,10-phenanthroline (lane 4) for 30 min at room temperature. Substrates were added and incubations proceeded for 24 h. Cleavage products were analyzed as described above. Mim (mimecan), Lum (lumican), Bgn (biglycan), Dec (decorin), FP (plasma fibronectin), Sup (*Leptospira* culture supernatant), Leptol. (leptolysin), Fen (1,10-phenanthroline).

Iniatially, to assess the minimum amount of leptolysin required for hydrolysis of SLRP, a dose-dependent assay using 0.025 – 0.2 µg of protease was performed. For the majority of substrates, efficient hydrolysis was observed with 0.1 μg of leptolysin (**Supplementary Figure 2**). Mimecam, also known as osteoglycin, and biglycan were completed hydrolyzed by the protease after 4h-incubation. Decorin was efficiently degraded as well, but lumican degradation required 24h-incubation. Limited proteolysis of fibronectin was also observed (**Figure 6A**). In all assays, *L. interrogans* serovar Kennewicki strain Fromm (LPF) supernatant was included as a positive control. The metal chelator 1,10-phenanthroline partially or totally abolished leptolysin proteolytic activity (**Figure 6B**). Our data suggest that leptolysin may, in fact, be one of the secreted *L. interrogans* metalloproteases capable of degrading components of the ECM.

### 3.6 *L. interrogans* leptolysin induces morphological changes in HK-2 cells

**Figure 7:**
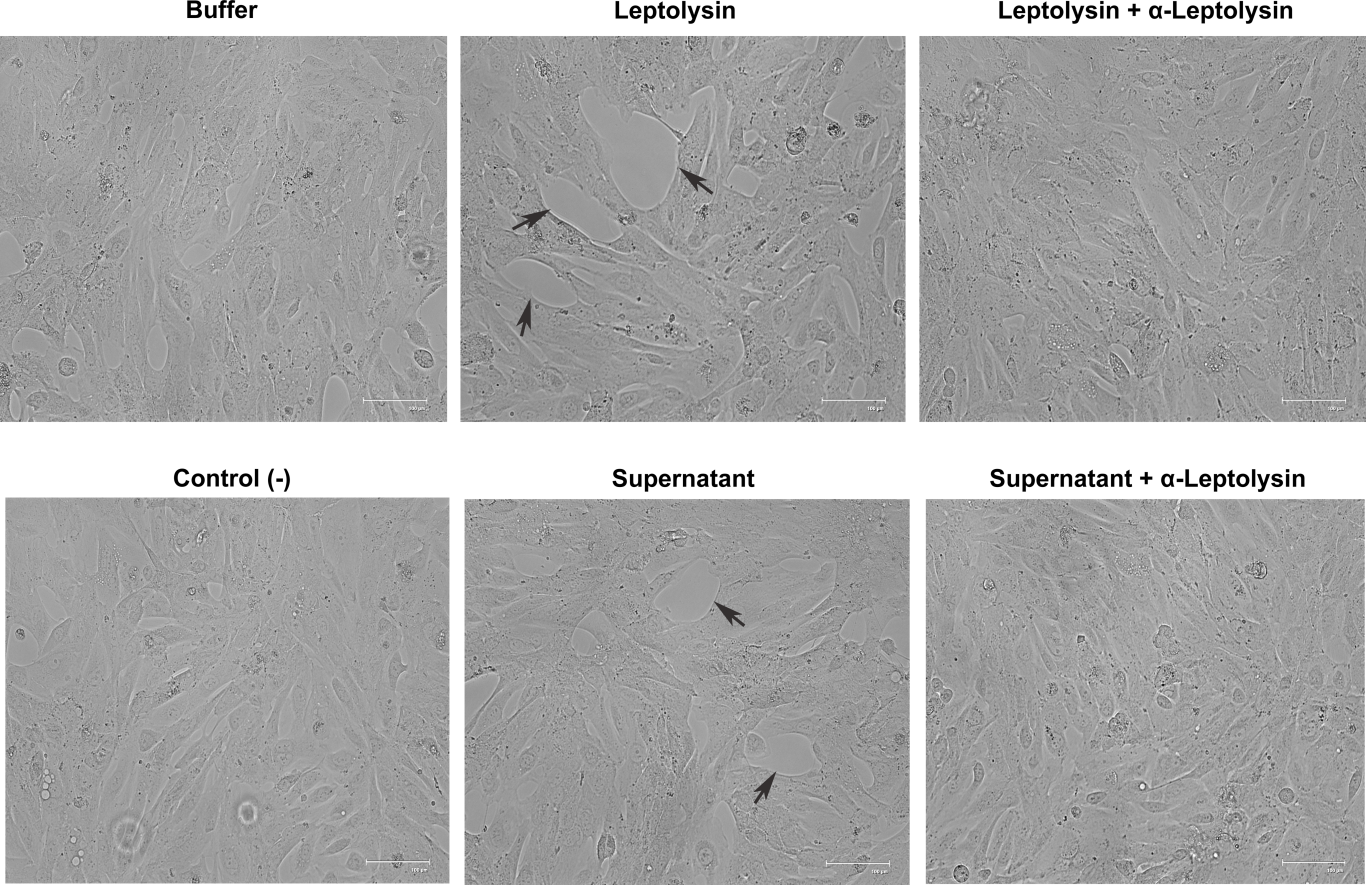
Morphological alterations induced by purified leptolysin and *L. interrogans* supernantant in renal cells. HK-2 cells were incubated for 24 h with leptolysin (0.5 µg) or Manilae L495 supernatant (5 µg) preincubated (or not) with leptolysin antiserum (1: 100). Negative controls included cells with culture medium (control -) and cells incubated with leptolysin buffer (buffer). Cells were visualized by light microscopy.

The effects of leptolysin on HK-2 cells, a proximal tubule epithelial cell line from normal adult human kidney, was then evaluated. Clearly, a dose-dependent effect of the protease on this kidney cell line could be observed. Detachment of adherent cells and morphological changes are evidently visible, as illustrated in **Supplementary Figure 3**. Cells were also incubated with *L. interrogans* serovar Manilae L495 supernatant. Although less remarkable than the phenotype observed for leptolysin treated cells, partial detachment of HK-2 cells was observed upon incubation with leptospiral supernatant, evidenced by empty regions pointed by the black arrows (**Figure 7**). Detachment was abolished when leplolysin or the supernatant were preincubated with leptolysin antiserum (**Figure 7**).

**Figure 8:**
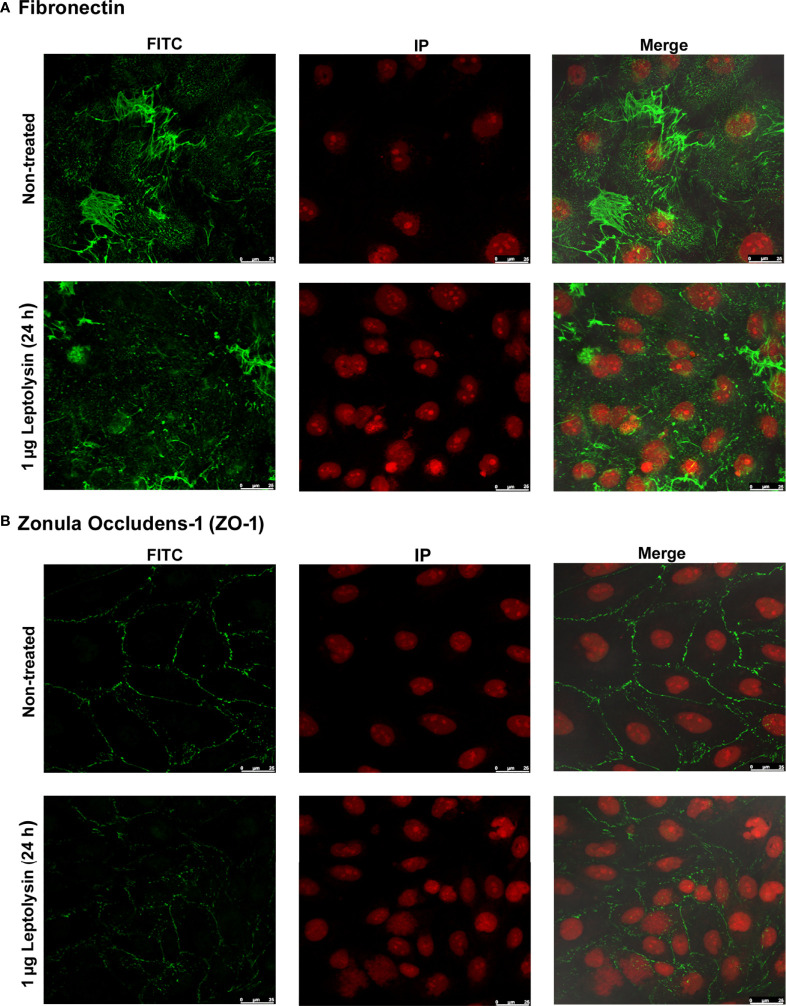
Effect of *L. interrogans* leptolysin on tight junction and ECM proteins in HK-2 cells detected by immunofluorescence microscopy. **(A)** Fibronectin and **(B)** Zonula occludens-1 (ZO-1) are shown in green. The nuclei are stained in red. Scale bars represent 25 μm.

Disturbances on ECM surrounding HK-2 cells, and on cell-cell adhesion molecules were preliminarily evaluated by immunofluorescence microscopy. In the leptolysin treated cells, the fibronectin fibrillar network pattern observed in untreated cells is less evident, assuming a more punctate morphology (**Figure 8A**). In addition, leptolysin induced a slight decrease in signal intensity of the tight junction molecule zonula occludens-1 (ZO-1), a peripheral membrane protein associated with the cell cytoplasmic surface (**Figure 8B**).

### 3.7 Proteolytic effects of *L. interrogans* leptolysin *in vivo*

**Figure 9:**
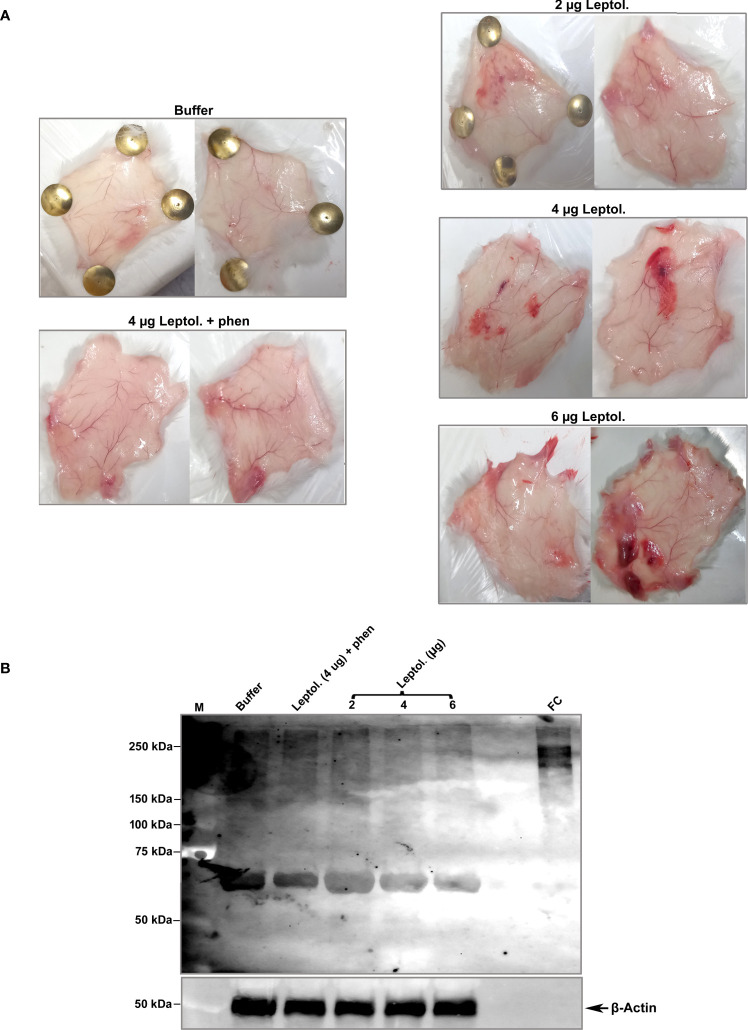
Degradation of fibronectin isolated from mouse skin injected with L. interrogans leptolysin. **(A)** Control skins (animals injected with buffer or with 4 µg of leptolysin + 1,10 phenanthroline) and skins injected with 2 - 4 µg leptolysin are shown. **(B)** Protein samples (4 µg) isolated from mouse skins were analyzed by Western blot with anti-fibronectin or anti-β actin as described in Materials and Methods.

To assess the effects of leptolysin on ECM *in vivo*, mice were intradermally injected in the dorsal skin with increasing amounts of the protease. After 4 h, animals were euthanized and skin fragments around the inoculation sites were removed, homogenized, and skin proteins were analysed by Western blot with antibodies to fibronectin, and abundant ECM component. Interestingly, hemorrhagic foci were observed in the dorsal skin of animals injected with 2 – 6 µg of recombinant leptolysin, but not in the control skins removed from mice inoculated with buffer or leptolysin (4 µg) preincubated with 1,10 – phenanthroline (**Figure 9A**). Hydrolysis of ECM and basement membrane components by certain metalloproteases is associated with hemorrhages, and may result either from the direct action of those enzymes or from tissue proteinases activated in the local inflammatory and hemorrhagic context (Asega et al., 2020).

Fibronectin isolated from mice skins migrated slightly different relative to human cellular fibronectin (FC), with an additional band of approximately 70 kDa that may correspond to the N-terminal fibronectin fragment harboring collagen/gelatin- and heparin-interacting domains (Dalton and Lemmon, 2021). Overall intensity of protein bands corresponding to fibronectin decreased in the hemorrhagic skins, notably in animals injected with 4 or 6 µg of recombinant leptolysin (**Figure 9B**, lanes 4 and 5). Thus, degradation or disruption of ECM integrity by direct or indirect action of leptolysin may lead to microvasculature destabilization and promote hemorrhage.

## 4 Discussion

Pappalysins belong to the metzincin clan of metalloproteases, characterized by the long zinc-binding consensus motif, HEXXHXXGXXH, and a methionine-containing Met-turn. Metzincins may unspecifically degrade an array of extracellular proteins, but may also display a more specific activity by targeting specific peptide bonds, thus contributing to regulatory and signaling physiological functions (Gomiz-Rüth, 2009; Brzdak et al., 2017). Tissue remodeling, embryonic development, inflammation, and immunity are largely controlled by the proteolytic activity of metzincins (Anthony et al., 2018).

In a previous work, exoproteomic analysis aiming to uncover *L. interrogans* - secreted proteases allowed identification of a few peptidases, among which a pappalysin-1 domain protein (da Silva et al., 2018), here named leptolysin. Like ulilysin and mirolysin, leptolysin is a short pappalysin. Despite the relatively low degree of sequence identity within the catalytic domain (ulilysin and mirolysin share 25% identity with leptolysin), the three metalloproteases present common recognition elements such as calcium, zinc and arginine-binding sites, conserved in pappalysins from bacteria, archea, fungi and mammals. Both ulilysin and mirolysin undergo autolytic activation mediated by calcium upon N-terminal removal of their prodomains. Curiously, native leptolysin, released in the extracellular medium, is detected as a single band of ~ 50 kDa in both saprophytic and pathogenic leptospires (**Figure 4**), leading us to consider that the protein does not undergo self-processing despite harboring a Ser-Arg site at position 230.

Overall, leptolysin catalytic domain is well conserved among *Leptospira* spp. However, residue variations in the vicinity of the catalytic site, observed in *Leptospira* species belonging to the P2 and S subclades, may affect protease stability, and eventually compromise binding to certain substrates. Alternatively, these variations may preclude binding to, and subsequent degradation of different substrates, which may not be necessarily present in the host. Further support to these assumptions will require experimental investigations to assess functionality and specificities of leptolysins produced by nonpathogenic *Leptospira*.

The biochemical analyses performed with *L. interrogans* leptolysin first aimed to determine the optimum values or conditions of three physical-chemical parameters - pH, temperature and cations effects - so that the attained results could be used in subsequent studies regarding its hydrolytic activities on FRET substrates. The pH of 8.0 was defined as optimal for the activity, and the highest velocity rate of Abz-GLARSNL-EDDnp hydrolysis was observed at 37°C. Lastly, leptolysin hydrolytic activity was positively influenced by monovalent cations, especially sodium and potassium, but negatively affected by divalent ions, such as magnesium and calcium. Inhibition of leptolysin by divalent cations may result from competition with the zinc ion present in the active site, causing its substitution and, thus, destabilizing the enzyme (Dudev and Lim, 2001; Rawlings et al., 2018). The use of a greater number of substrates for a more accurate primary specificity study will be necessary, but the preliminary results presented here, using the cleavage points attained on five FRET peptides, which are used by our group to study metalloproteases (Cajado-Carvalho et al., 2019), provided relevant information on leptolysin preferences for substrate hydrolysis. A high preference for arginine at the S1 subsite of leptolysin was observed. However, arginine residues were also found in the P1’ position. The S3 subsite showed a good interaction with hydrophobic amino acids, such as leucine and phenylalanine, while glutamine and leucine were found in the P2 position. Thus, the specificity of leptolysin for substrate hydrolysis may be different from those reported for ulilysin, mirolysin, human PAPP-A and PAPP-A2, which show preferences for arginine and lysine residues in the P1’ position of their substrates, but substrates with phenylalanine and asparagine in this position are reported (Conover, 1995; Boldt et al., 2001; Overgaard et al., 2001; Laursen et al., 2002a; Laursen et al., 2002b; Tallant et al., 2006; Tallant et al., 2007; Koneru et al., 2017) Differences between the primary and tertiary structures observed between leptolysin and the other analyzed metalloproteases may reflect differences in primary specificities.

The SLRPs, including decorin, biglycan, mimecan, and lumican, are ubiquitously found in the ECM and are engaged in matrix organization and regulation of cell growth and signaling (Appunni et al., 2019). As ECM and plasma molecules are susceptible to degradation by leptospiral metalloproteases released in the extracellular medium (da Silva et al., 2018), *L. interrogans* leptolysin was then evaluated for the capacity to hydrolize proteoglycans and plasma fibronectin. All proteinaceous substrates were efficiently degraded over time, confirming leptolysin´s broad-spectrum activity *in vitro*. *In vivo* degradation of mouse skin derived fibronectin was also observed in animals injected with leptolysin. Hemorrhagic foci due to destabilization of the local microvasculature were found close to the inoculation sites, and may be associated with ECM disarray and vascular endothelium glycocalyx damage.

It is plausible to assume that leptospiral proteases play an important role in all stages of the infectious process: *i*) during bacterial entry and dissemination they may contribute to tissue degradation; *ii*) by degrading complement effector proteins in the bloodstream, secreted proteases may allow persistence, and, finally, *iii*) they may cause tissue damage by inducing inflammation, vascular injury and haemorrhages (Murray, 2015). Therefore, the characterization of leptospiral proteases, their substrates and mechanisms of action may potentially help developing therapeutics to mitigate damage caused by this spirochete. Interestingly, some of the features found in leptolysin are shared by the *Treponema pallidum* protease pallilysin (Tp0751), described as having both adhesive and proteolytic activities (Houston et al., 2011; Houston et al., 2012; Lithgow et al., 2020). Tp0751 is a zinc-dependent metalloprotease harboring conserved histidines involved in metal binding (**H**EXX**H**). In association with *T. pallidum* Tp0750, which contains a Von Willebrand factor type A domain, it has been reported to promote fibrinolysis and ECM degradation (Houston et al., 2012; Houston et al., 2014). However, leptolysin is secreted extracellularly whereas pallilysin was recently shown to reside within the periplasmic space (Luthra et al., 2020).

*L. interrogans* leptolysin´s cytopathic effects were also evaluated on HK-2 cells, a proximal tubule epithelial cell line from normal adult human kidney. A dose-dependent effect was clearly evident upon treatment. Cell detachment could be partially attributed to fibronectin degradation, but other ECM macromolecules must have been affected by leptolysin as well. In order to evaluate whether this particular protease would target cell-cell junctions, we selected one of the several molecules that make up tight junctions: zonula occludens-1 (ZO-1). The pattern of ZO-1 in treated cells appears less intense compared to the control, but it is uncertain if this slightly altered morphology results from the direct action of the protease or if it stems from ECM degradation around the cells. Interestingly, in human dermal lymphatic endothelial cells (HDLEC) infected with *L. interrogans* serovar Copenhageni the fluorescence signal of ZO-1 was relocalized intracellularly (Sato and Coburn, 2017). In renal proximal tubule epithelial cells (RPTECs) *L. interrogans* infection led to disassembly of the apical junctional complex causing mislocalization of ZO-1 and other AJC proteins, as well as cytoskeletal rearrangement (Sebastián et al., 2021). Screening a larger panel of ECM and cell-cell junction proteins will help elucidating the primary targets of leptolysin, and its role in *Leptospira* dissemination within the host.

Here we present a first characterization of *L. interrogans* leptolysin, a pappalysin that is highly conserved among *Leptospira* species belonging to the subclade P1. Leptolysin´s broad-spectrum proteolytic capacity justifies ongoing *in vivo* studies that may provide further evidence of the involvement of this metalloprotease in the pathogenesis of leptospirosis, through inactivation of host proteins that may impact tissue integrity, coagulation and complement functions.

## Data availability statement

The original contributions presented in the study are included in the article/**Supplementary Material**. Further inquiries can be directed to the corresponding authors.

## Ethics statement

The animal study was reviewed and approved by the Committee on Ethics of Instituto Butantan (protocol # 9452010316 approved in the meeting of 03/16/2016, and protocol #1033050321 approved in the meeting of 03/30/2021) which are in agreement with Law 11.794, of October 8 2008, Decree 6899, of July 15, 2009, with the rules issued by the National Council for Control of Animal Experimentation (CONCEA).

## Author contributions

Conceptualization: AB and DC. Formal analysis: AB, FP, and RR-d-S. Funding acquisition: AB, MH, and LI. Investigation: DC, CS, LP, RC-C, GS, FP, and RR-d-S. Methodology: DC, CS, LP, RC-C, GS, FC, RR-d-S. Resources: MH, LI, LM, FP, and AB. Supervision: AB, LM, LI, MH, and FP. Visualization: DC, CC, LP, RC-C, LM, GS, MH, LI, FC, FP, RR-d-S, and ASB. Writing – original draft: AB. Writing - review & editing: DC, CC, LP, RC-C, LM, GS, MH, LI, FC, FP, RR-d-S, and AB. All authors contributed to the article and approved the submitted version.

## Funding

This work was supported by Fundação de Amparo à Pesquisa do Estado de São Paulo (FAPESP), grants 2018/12896-2, 2019/22706-9 and 2017/12924-3, and was financed in part by the Coordenação de Aperfeiçoamento de Pessoal de Nível Superior – Brasil (CAPES) – Finance Code 001.

## Acknowledgments

We would like to thank the “Laboratório de Biologia Celular” from Butantan Institute and the technician Alexsander Seixas de Souza for the services provided on the Confocal Microscope Leica TCS SP8 (Project 175 FINEP—IBUINFRA grant 01.12.0175.00 coordinated by Dr. Carlos Jared). We also thank Dr. Daniel Pimenta from the Biochemistry and Biophysics Laboratory of the Butantan Institute for the mass spectrometry analysis, and Dr. Elsio Wunder from Yale School of Public Health, New Haven, USA, for providing *L. interrogans* serovar Manilae strain L495.

## Conflict of interest

The authors declare that the research was conducted in the absence of any commercial or financial relationships that could be construed as a potential conflict of interest.

## Publisher’s note

All claims expressed in this article are solely those of the authors and do not necessarily represent those of their affiliated organizations, or those of the publisher, the editors and the reviewers. Any product that may be evaluated in this article, or claim that may be made by its manufacturer, is not guaranteed or endorsed by the publisher.

## Supplementary material

Supplementary Figure 1 Preliminary study of primary specificities of leptolysin. Cleavage points on FRET substrates present in **Table 2** using the iceLogo software (Colaert et al., 2009).

Supplementary Figure 2 Dose-dependent degradation of proteoglycans and plasma fibronectin by *L. interrogans* leptolysin. Mimecan, lumican, biglycan, decorin (0.5 μg), and plasma fibronectin (5 μg) were incubated with leptolysin (25 – 200 ng) at 37°C for 24 h. Cleavage products were subjected to SDS- polyacrylamide gel under reducing conditions, transferred to nitrocellulose membranes, and probed with specific antibodies, or the gel was silver stained (FP), as described in Materials and Methods. Mim (mimecan), Lum (lumican), Bgn (biglycan), Dec (decorin), FP (plasma fibronectin).

Supplementary Figure 3 Morphological alterations induced by *L. interrogans* leptolysin in renal cells. HK-2 cells were incubated with 0.1 µg, 0.5 µg or 1 µg of recombinant leptolysin for 24 h. Analysis was performed under a light microscope. As a control, cells were incubated with buffer. Magnification: 400 x.
