# Supplementary figures and images for: Leptolysin, a Leptospira secreted metalloprotease of the pappalysin family with broad-spectrum activity
Source: Front Cell Infect Microbiol. 2022 Aug 23;12:966370. doi: 10.3389/fcimb.2022.966370 (PMC9445424; doi:10.3389/fcimb.2022.966370)

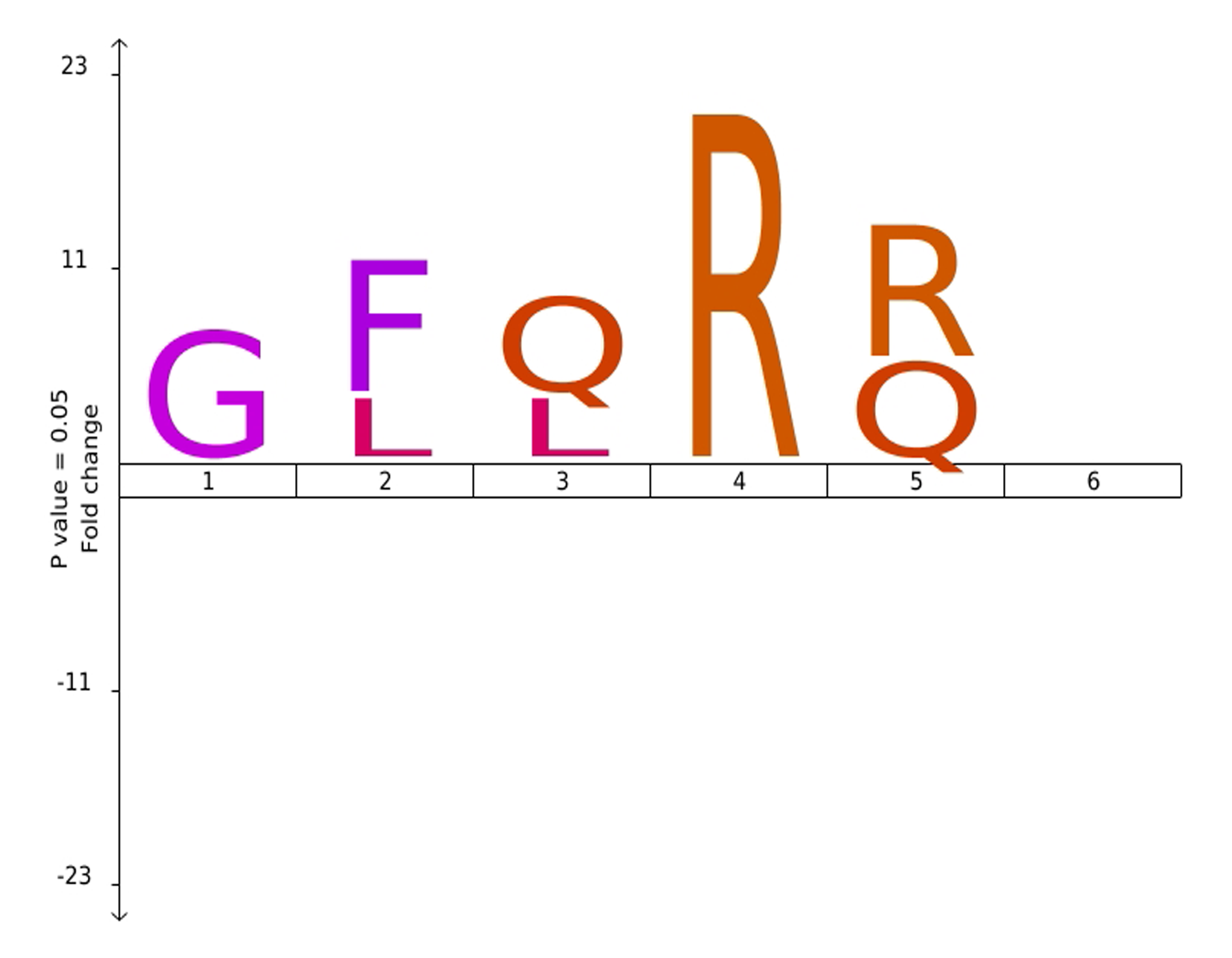

Supplement: Supplementary Figure 1 — Preliminary study of primary specificities of leptolysin. Cleavage points on FRET substrates present in Table 2 using the iceLogo software (Colaert et al., 2009). [file Image_1.tif]

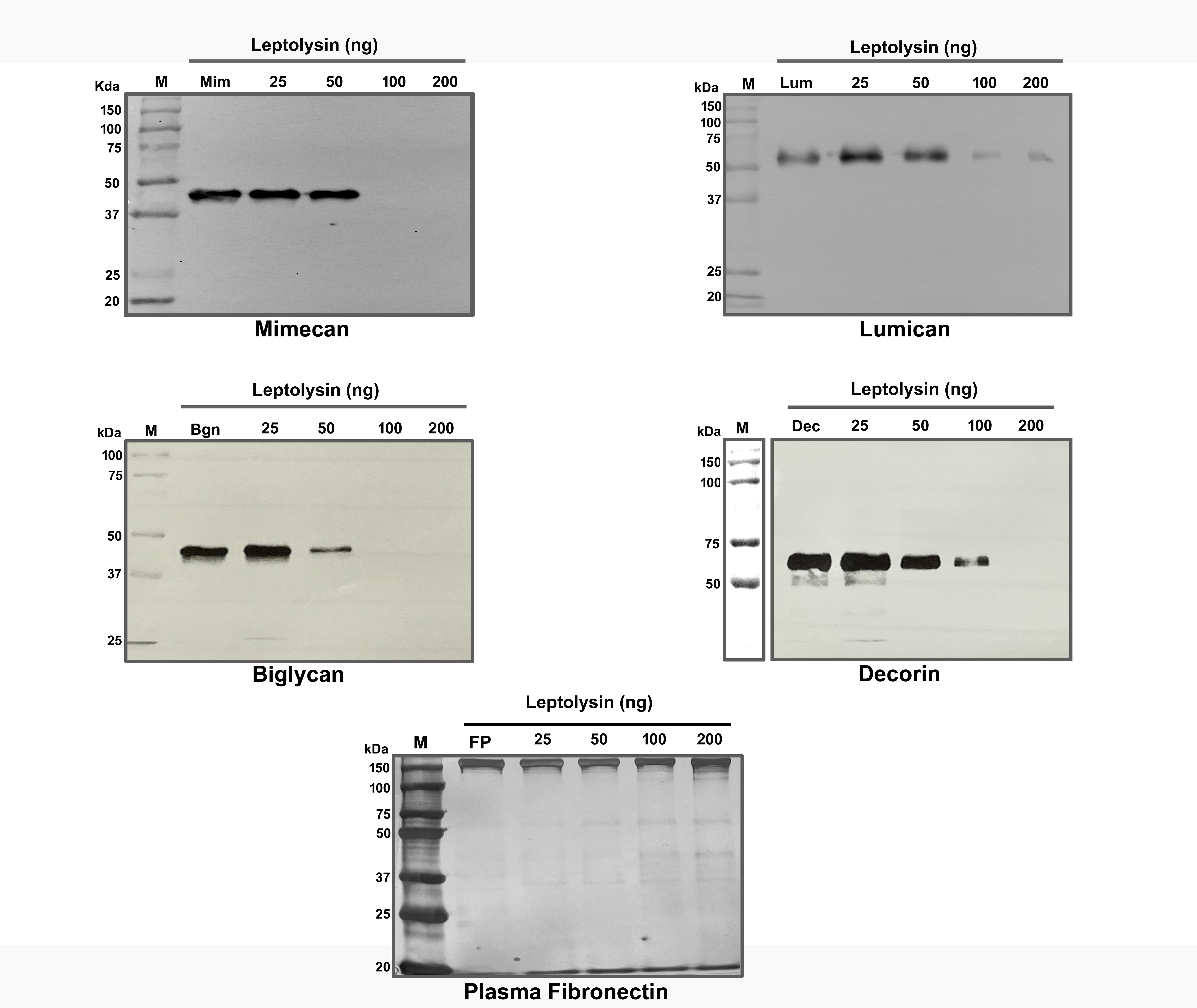

Supplement: Supplementary Figure 2 — Dose-dependent degradation of proteoglycans and plasma fibronectin by L. interrogans leptolysin. Mimecan, lumican, biglycan, decorin (0.5 μg), and plasma fibronectin (5 μg) were incubated with leptolysin (25 – 200 ng) at 37°C for 24 h. Cleavage products were subjected to SDS- polyacrylamide gel under reducing conditions, transferred to nitrocellulose membranes, and probed with specific antibodies, or the gel was silver stained (FP), as described in Materials and Methods. Mim (mimecan), Lum (lumican), Bgn (biglycan), Dec (decorin), FP (plasma fibronectin). [file Image_2.tif]

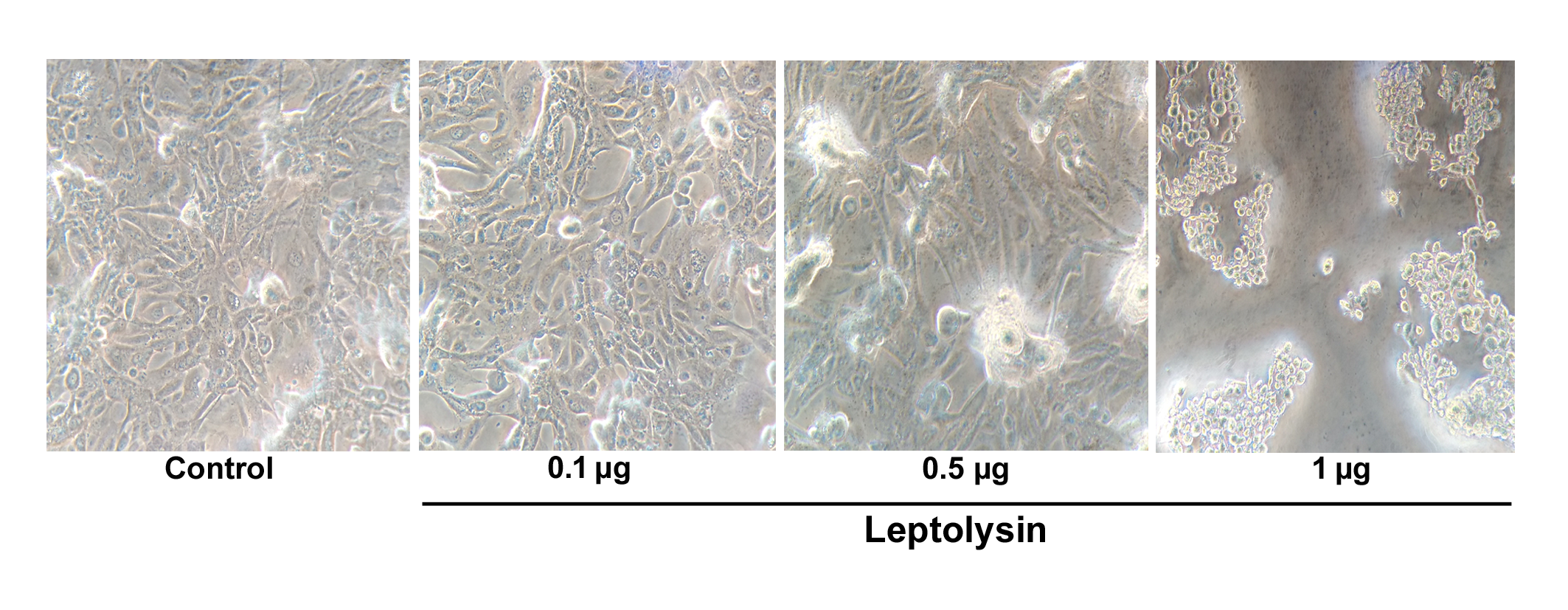

Supplement: Supplementary Figure 3 — Morphological alterations induced by L. interrogans leptolysin in renal cells. HK-2 cells were incubated with 0.1 µg, 0.5 µg or 1 µg of recombinant leptolysin for 24 h. Analysis was performed under a light microscope. As a control, cells were incubated with buffer. Magnification: 400 x. [file Image_3.tif]
